# Molecular Paleontology Meets Drug Discovery: The Case for De-extinct Antimicrobials

**DOI:** 10.1021/acsomega.5c05530

**Published:** 2025-09-10

**Authors:** Rumiana Tenchov, Qiongqiong Angela Zhou

**Affiliations:** 2645 CAS, a Division of the American Chemical Society, Columbus, Ohio 43210, United States

## Abstract

The rise of antibiotic resistance has necessitated the exploration of unconventional sources of novel antimicrobial agents. One emerging novel frontier is “de-extinct” molecules—bioactive peptides, antibiotics, and other bioactive agents reconstructed from ancient or extinct organisms—an innovative convergence of paleogenomics, paleoproteomics, and synthetic biology. Recent advances in high-throughput DNA sequencing, mass spectrometry, and computational biology have enabled scientists to recover and analyze genetic and protein sequences from long-extinct species, offering unprecedented insights into evolutionary biology and potential applications in medicine, biotechnology, and conservation, including the successful regeneration of antimicrobial molecules from several extinct organisms. While paleogenomics provides the blueprint for reconstructing extinct genomes, paleoproteomics offers complementary insights into gene expression, protein function, and post-translational modifications that are often lost in DNA-based studies. These approaches can yield proteins and metabolites that have been lost to evolution, offering a new reservoir of bioactive compounds that could be used for new strategies in medicine, biotechnology, and synthetic biology. In this report we explore data from the CAS Content Collection to outline the current landscape and research progress in the emerging area of molecular de-extinction, to identify key developing concepts and challenges, and to identify successfully revived de-extinct antimicrobials. We outline the technical approaches to their revival in an effort to understand how this highly innovative strategy helps combat modern multidrug-resistant pathogens as well as the challenges and ethical considerations in deploying ancient molecules.

## Graphical abstract

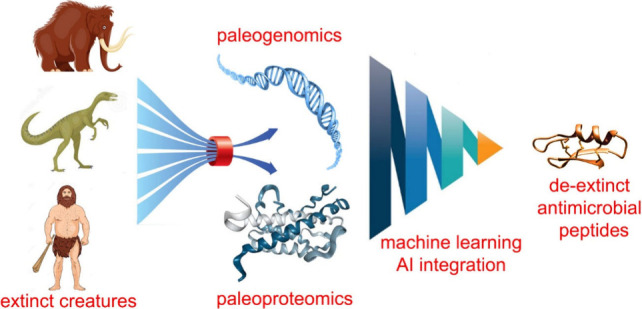

## 1 Introduction

The concept of de-extinction has long captured scientific and public imagination, traditionally framed around the revival of extinct species such as the woolly mammoth or the passenger pigeon.^1^ However, a more immediate and tractable application lies in molecular de-extinction—the selective resurrection of extinct genes, proteins, or metabolic pathways rather than whole organisms.^2–5^ This emerging field leverages two primary scientific disciplines: paleogenomics, the study of ancient DNA (aDNA), and paleoproteomics, the analysis of ancient proteins preserved in fossilized and subfossil remains. These approaches allow scientists to mine evolutionary history for novel bioactive compounds that could bring new strategies to medicine, biotechnology, and synthetic biology. At the same time, reviving ancient genes can provide significant knowledge on evolutionary history.^6^ Notably, a study of immune genes of Neanderthals rationalized our susceptibility to emerging infectious diseases such as COVID-19: a gene cluster on chromosome 3, identified as the major genetic risk factor for respiratory failure after infection with SARS-CoV-2, was conferred by a genomic segment inherited from Neanderthals and has been found to be carried by ∼50% of people in South Asia and ∼16% of people in Europe.^7–9^

Paleogenomics has advanced our understanding of evolutionary biology, enabling the sequencing of genomes from species that disappeared thousands or even millions of years ago. However, DNA is not the sole bearer of biological information; proteins, which are more chemically stable in certain environments, provide critical complementary data on gene expression, structural biology, and functional biochemistry.^10^ While aDNA degrades rapidly due to hydrolysis and oxidation, proteins—particularly those with stable secondary structures—can persist for much longer periods in fossils, permafrost, and archeological specimens. For example, fragments of collagen protein have been successfully sequenced from the bones of a 68-million-year-old *Tyrannosaurus rex* and a 600,000-year-old mastodon.^11,12^ Paleoproteomics thus bridges gaps left by degraded or incomplete genomes, offering insights into extinct species’ physiology, immune responses, and biochemical adaptations.

Recent technological advancements have propelled molecular de-extinction from theoretical speculation to experimental reality. Next-generation sequencing (NGS) and third-generation long-read sequencing have dramatically improved the recovery of highly fragmented aDNA, while high-resolution mass spectrometry (MS) and bioinformatic protein modeling now allow researchers to reconstruct ancient protein sequences and predict their functions.^13–15^ Case studies, such as the resurrection of a 5,000-year-old bacterial β-lactamase enzyme and the functional analysis of Neanderthal immune-related proteins, demonstrate the potential of these approaches in biotechnology and medicine. With progress in computational biology and artificial intelligence, the identification of favorable molecules has transitioned from a largely random process to a more deliberate, data-driven methodology, where researchers can actively target specific molecular characteristics based on extensive data analysis to predict and validate their potential efficacy (Figure 1).^16–19^

**Figure 1:**
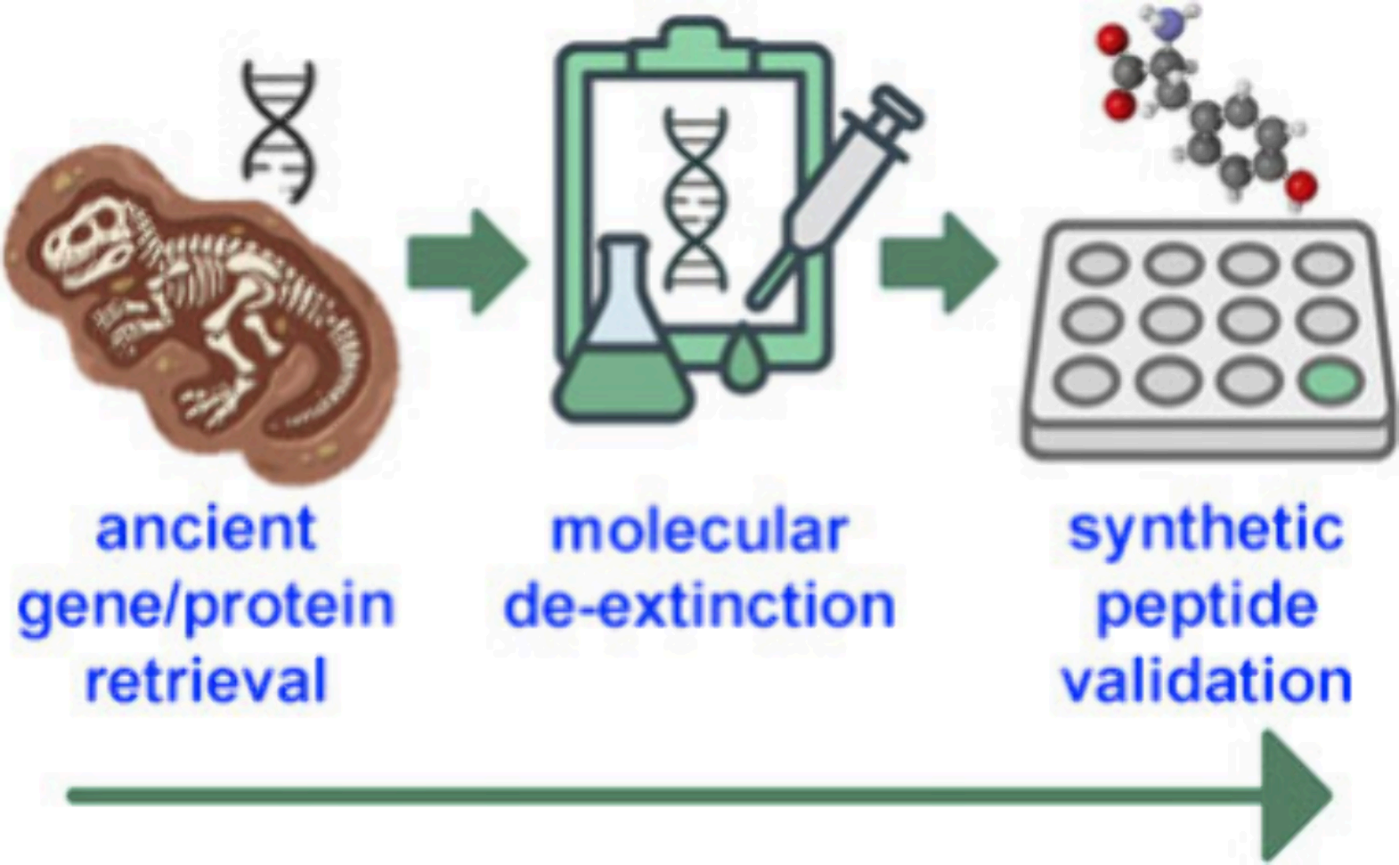
Scheme of the workflow for new peptide validations via molecular de-extinction.

Among the diverse array of molecules identified through proteomics or genomics, antimicrobial peptides (AMPs) are particularly noteworthy. AMPs have been integral to the defense mechanisms of animals, evolving over millions of years to safeguard hosts against a variety of pathogens, thereby ensuring survival in ancient environments.^20^ They continue to play vital roles in the innate immune systems of various organisms, combating microorganisms. However, it is important to emphasize the disparity between the number of discovered antimicrobial peptides and those that successfully progress through clinical trials and ultimately reach the market; while many AMPs show promise in lab settings, several challenges hinder their translation to effective treatments. These challenges include instability, potential toxicity, and difficulties with delivery and bioavailability.^21^ The progress of artificial intelligence and molecular de-extinction presents a worthwhile possibility to provide precise *in silico* estimates, along with uncovering new antimicrobials, thus accelerating the effort to address the antibiotic resistance crisis.

Genome editing and synthetic biology are revolutionizing de-extinction, offering unprecedented tools to restore lost biodiversity. However, significant challenges remain. DNA degradation limits the recovery of complete genomes, while post-mortem protein modifications complicate the accurate reconstruction of ancient proteomes. Additionally, even successfully resurrected biomolecules may not function as expected in modern biological systems due to differences in cellular environments, epigenetic regulation, and post-translational processing. Beyond technical hurdles, ethical concerns—such as ecological risks from reintroduced genetic elements, biosecurity threats posed by resurrected pathogens, and the moral implications of “playing God”—demand careful consideration.

### Case Studies

The woolly mammoth project (Colossal Biosciences): The approach included CRISPR editing of Asian elephant iPSCs to introduce mammoth traits. The goal of the project was to create a cold-resistant elephant-mammoth hybrid to restore Arctic ecosystems.^22^

The passenger pigeon project (Revive & Restore): The approach included editing band-tailed pigeon genomes to restore extinct behavioral and morphological traits. The major challenge was reconstructing complex social behaviors from genetic data.^23^

The thylacine project (TIGRR Lab): The approach included using CRISPR to modify fat-tailed dunnart genomes with thylacine DNA. The unique hurdle was the marsupial reproductive biology which complicates surrogate pregnancy.^24^

Here, a word of caution regarding de-extinction of organisms is needed. It is widely recognized that de-extinction, particularly through genetic engineering and cloning, results in organisms that are not identical with the extinct species, but rather hybrids or chimeras. This raises certain ethical dilemmas.^25,26^ Some argue that de-extinction, especially when involving significant genetic modification, creates organisms that are not truly members of the extinct species but rather “functional equivalents” or ecological proxies. De-extinction attempts, particularly through cloning, have raised concerns about the potential for suffering in the resulting organisms, as seen with the bucardo cloned and born in 2003 with deformed lungs.^27^ De-extinction would create genetically modified species, potentially leading to unforeseen environmental consequences. These new species could interfere with and compete with existing species, acting as invasive species.^28^ De-extinction is viewed by some as hubris, an overstepping of human boundaries by interfering with natural processes, especially death and creation.^29^ These issues require a careful, case-by-case ethical evaluation of any de-extinction project.

This paper examines the current state of molecular de-extinction research, focusing on the synergies between paleogenomic and paleoproteomic approaches. We explore data from the CAS Content Collection,^30^ the largest human-curated repository of scientific information, to outline the current landscape and research progress in molecular de-extinction, to identify key emerging concepts and challenges, successfully revived de-extinct antimicrobials, and the technical approaches to their revival, in an effort to understand how this highly innovative strategy helps combat modern multidrug-resistant pathogens. We evaluate case studies where these methods have been successfully applied, discuss persistent limitations, challenges, and ethical considerations in deploying ancient molecules, and explore future directions for research.

## 2 CAS Content Collection Landscape

A combined search on de-extinction/paleogenomic/paleoproteomic identifies 450 documents, exclusively journal articles, with only one patent (WO2025054593).^31^ Dominating are the paleogenomics articles, the number of which is consistently growing. Fourteen documents published in the past three years (2023–2025) are specifically focused on molecular de-extinction delineating it as a novel, inventive research area (Figure 2).^2,3,13,31–39^ The peak of the de-extinction-related documents in 2017 is related to The Hastings Center for Bioethics^40^ publishing a special issue “Recreating the Wild: De-Extinction, Technology, and the Ethics of Conservation”.^41^

**Figure 2:**
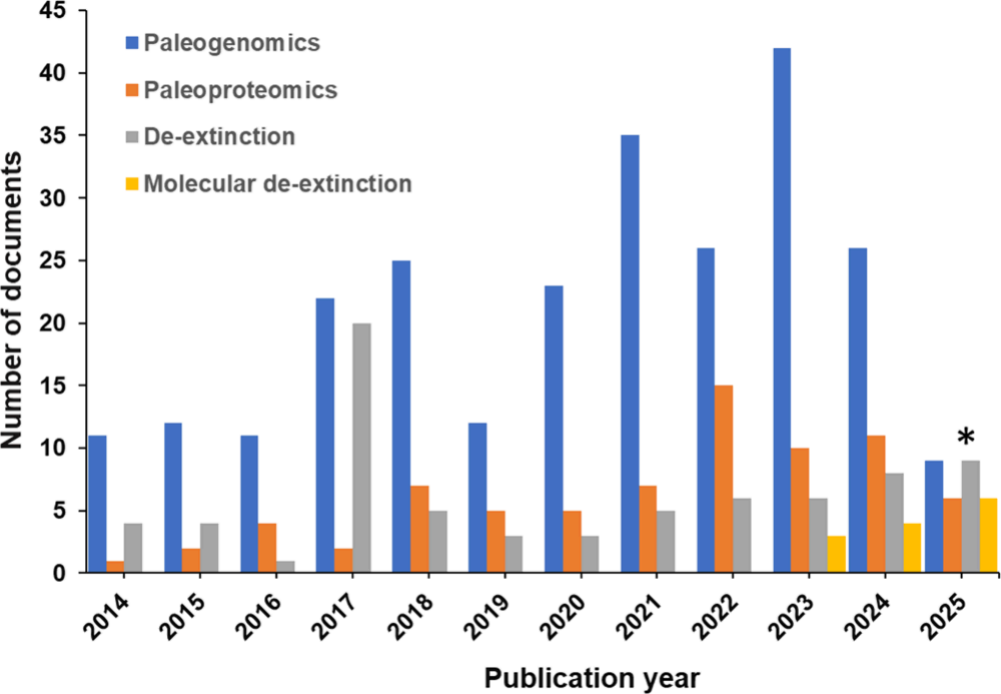
Yearly growth of the number of documents including the major concepts related to molecular de-extinction field, as found in the CAS Content Collection (asterisk indicates data for 2025 is partial—only until March 2025).

We examined the assortment of essential concepts related to de-extinction in the published documents found in the CAS Content Collection (Figure S1 in the Supporting Information). **Paleogenomics**, the study of ancient DNA (aDNA), has emerged as the most widely explored and foundational concept in de-extinction—the process of resurrecting extinct species or engineering functional equivalents.^42–44^ While other approaches (e.g., back-breeding or cloning) contribute to de-extinction, paleogenomics provides the genetic roadmap necessary for precise species revival. By recovering, sequencing, and analyzing genetic material from extinct species, researchers can reconstruct lost genomes, identify key functional adaptations, and engineer living proxies through genome editing. The advances in high-throughput sequencing, CRISPR-Cas9 gene editing, and synthetic biology are converging to make substances and species revival a tangible—albeit complex—scientific endeavor.

**Antimicrobials and antibiotics** are the subject of the most molecular de-extinction-related documents in the recent three years. Recent advances in paleogenomics have revealed unexpected connections between molecular de-extinction research and antimicrobial discovery. Cutting-edge work in species resurrection is driving innovations in combating antibiotic-resistant pathogens. Three key intersections are most noteworthy: (1) ancient antimicrobial peptide discovery through paleogenomics, (2) CRISPR-based antimicrobial strategies developed from de-extinction tools, and (3) novel antibiotic discovery from resurrected microbial communities.^2^ These converging fields represent rapidly growing areas in modern biotechnology, offering solutions to both biodiversity loss and global health crises.

Another concept which recently coappears with de-extinction is **paleopathology**, the study of ancient diseases.^45^ It is traditionally focused on understanding health in past populations through skeletal and mummified remains. However, recent advances in paleogenomics, paleoproteomics, and bioinformatics have expanded its applications into modern drug discovery.^46^ Paleopathology, combined with molecular archeology, offers a unique solution: studying ancient diseases and past medical practices to identify lost therapeutic compounds and evolutionary defense mechanisms. By analyzing ancient pathogens, human immune responses, and extinct medicinal compounds, researchers are uncovering novel therapeutic strategies to combat antibiotic resistance, chronic diseases, and emerging infections.^47,48^ Furthermore, integrating evolutionary biology with paleopathology offers a better understanding of selective pressures that affected cancer susceptibility in extinct species and identify potential mechanisms of tumor resistance.^49^

**Neanderthals**, another concept frequently coappearing with de-extinction, are an extinct group of archaic humans who occupied western Eurasia during the Late Pleistocene.^50–52^ Genome-scale data have been made available for the skeletal remains from 14 archeological sites spanning Neanderthal history across large parts of their identified geographical areas.^50^

## 3 Paleoproteomics

### Ancient Antimicrobial Peptides Enabled by Deep-Learning

The traditional methods of antibiotic discovery such as natural product screening, chemical synthesis, and high-throughput screening have undoubtedly been successful; however, they are limited by the scope of natural diversity and chemical libraries. As resistant bacteria keep evolving, the demand for new antibiotics becomes increasingly important, demanding innovative approaches to discovery. A noteworthy approach included searching for altered biosynthesis pathways (and hence modified antibiotics) based on resistance prevalence and then synthesizing the potential molecules to avoid the need to isolate the agent itself.^53,54^ Deep learning, a subset of artificial intelligence (AI), with its power to analyze massive data sets, and molecular de-extinction, which can resurrect ancient genes and proteins, provide a paradigm shift in the pursuit of novel antibiotics. By combining these approaches, it becomes possible to explore unique, previously unavailable chemical areas and discover new bioactive compounds.

Thus, by mining all available proteomes of extinct organisms by applying a deep mining algorithm, APEX, scientists were able to discover new antibiotic peptides.^4,32^ Deep-learning models for proteolytic site prediction were trained to project the antimicrobial activity by a large range of proteases in the proteomes of extinct organisms (so-called ‘extinctome’). A large collection of sequences not found in extant organisms were predicted to exhibit broad-spectrum antimicrobial activity. Sixty-nine peptides were synthesized, and their activity against bacterial pathogens was experimentally verified. Examples of active antimicrobial peptides from various extinct organisms are shown in Table 1. Furthermore, several pairs of peptides from the same extinct organism exhibited particularly strong synergistic interactions against pathogens such as *A. baumannii* and *P. aeruginosa*, with fractional inhibitory concentration (FIC) index values (numerical values used to assess the interaction between two or more antimicrobial agents when combined in a test, typically calculated by dividing the minimum inhibitory concentration (MIC) of each drug when used in combination by its MIC when used alone, and then summing the results^55,56^ ) as low as 0.38 for *A. baumannii*.^31,32^ For the combination of Equusin-1 and Equusin-3, the MICs decreased by 64 times (from 4 μmol L^–1^ to 62.5 nmol L^–1^), reaching submicromolar concentrations that are comparable to the MICs of most potent antibiotics.^57^

**Table 1:** Examples of Active Antimicrobial Peptides from Various Extinct Organisms as Identified by the APEX Deep Learning Algorithm

Peptide/*CAS RN*	Extinct organism	Parent protein	Peptide sequence	MIC
Hydrodamin-1	Steller’s sea cow (*Hydrodamalis gigas*)	Endothelial differentiation gene 1	LYCRIYSLVRARG RRLTFRKNISK	4 μmol L^–1^ (*A. baumannii, E. faecium*)
***3078251–51–8***				
Megalocerin-1	Giant elk (*Megaloceros gigantescus*)	Cytochrome c oxidase subunit 3	LIVCFFRQLKFHF	8 μmol L^–1^ (*A. baumannii, E. faecium*)
***3078251–56–3***				
Mylodonin-2	Giant sloth (*Mylodon darwinii*)	Apolipoprotein B	KRKRGLKLATALS LNNKF	32 μmol L^–1^ (*E. coli*)
***3078251–68–7***				
Elephasin-2	Straight-tusked elephant (*Elephas antiquus*)	ATP synthase F0 subunit 8	IFLHLKILKIIRLL	1 μmol L^–1^ (*A. baumannii, S. aureus, E. faecium*)
***3078251–59–6***				
Mammuthusin-2	Siberian woolly mammoth (*Mammuthus primigenius*)	Melanocortin-1 receptor	RACLHARSIARLHK RWRPVHQGLGLK	32 μmol L^–1^ (*A. baumannii, E. faecium*)
***3078251–49–4***				
Equusin-1	Grant’s zebra (*Equus quagga boehmi*)	Natural resistance-associated macrophage protein 1	FLKLRWSRFARVLL	1 μmol L^–1^ (*E. faecium*) 4 μmol L^–1^ (*A. baumannii, E. coli, P. aeruginosa*)
***3078251–35–8***				
Equusin-2	Grant’s zebra (*Equus quagga boehmi*)	Abnormal spindle-like microcephaly associated protein	KIYKKLSTPPFTL NIRTLPKVKFPK	8 μmol L^–1^ (*A. baumannii*)
***3078251–48–3***				

Remarkably, top compounds, including Mammuthusin-2, Elephasin-2, Hydrodamin-1, Mylodonin-2, and Megalocerin-1, exhibited potential anti-infective activity in mice with skin abscess or thigh infections (Table 1).^32^ The results obtained for the more active peptides tested in skin abscess infection model (Elephasin-2 and Mylodonin-2) indicated antibacterial activity comparable to that of the widely used antibiotic polymyxin B. Similarly, Mylodonin-2 and Elephasin-2 exhibited comparable anti-infective efficacy to polymyxin B when using a murine deep thigh infection model, thus underscoring the potential of molecular de-extinction as a successful approach for antibiotic discovery.^32^

The commercial potential of the revived ancient peptides is reflected in a recent patent (WO2025054593) disclosing the methods for identifying antimicrobial peptides derived from extinct proteomes using a multitask deep learning algorithm, APEX, along with the identified 41 antimicrobial peptides, their synergy and mechanism of action, and the methods of treating microbial infections with these antimicrobial peptides.^31^

Furthermore, the created machine learning tool, panCleave random forest model for proteome-wide cleavage site prediction, was applied to power the exploration a pan-protease cleavage site classifier to perform computational proteolysis—an *in silico* digestion of human proteins.^2,3,18,36^ So, machine learning approaches were used for molecular de-extinction, whereby the proteomes of our closest relatives, the archaic humans Neanderthals and Denisovans, were mined, and several encrypted peptide antibiotics were resurrected which displayed antimicrobial activity *in vitro* and in preclinical mouse models (Table 2).^2^

**Table 2:** Examples of Active Antimicrobial Peptides from Archaic Humans as Identified by the panCleave Machine Learning Tool

Protein fragment ID/*CAS RN*	Source extinct organism	Sequence	MIC
PDB6I34D-ALQ29	Neanderthal glycine decarboxylase protein	ALQLCYRH NKRRKFFV DPRCHPQTI AVVQ	32 μmol L^–1^ (*P. aeruginosa*), 128 μmol L^–1^ (*E. coli*)
***3086295–62–4***			
A0A0S2IB02-AYT38	Denisovan transmembrane protein	AYTTWNIL SSAGSFISL TAVMLMIF MIWEAFAS KRKVL	128 μmol L^–1^ (*P. aeruginosa*)
***3086350–67–3***			
A0A343EQH0-NVK38	Denisovan transmembrane protein	NVKMKWQ FEHTKPTPF LPTLITLTT LLLPISPFM LMIL	128 μmol L^–1^ (*P. aeruginosa*)
***3086350–66–2***			
A0A343AZS4-FMA25	Denisovan NADH-ubiquinone oxidoreductase	FMAEYTNII MMNTLTTT IFLGTTYN	128 μmol L^–1^ (*A. baumannii*)
***3086295–65–7***			
A0A343EQH4-LAM11	Denisovan NADH-ubiquinone oxidoreductase	LAMVIPLW AGA	128 μmol L^–1^ (*A. baumannii*)
***3086295–64–6***			
A0A384E0N4-DLI09	Neanderthal adenylosuccinate lyase	DLIERIQAD	128 μmol L^–1^ (*A. baumannii, S. aureus*)
***3086295–63–5***			

Noteworthy, while the identification of potential AMPs in ancient organisms provides valuable leads for antibiotic discovery, it is crucial to recognize that the predicted antimicrobial activity in a lab setting does not automatically equate to that being their primary or sole function in the ancient organisms physiology. Finding a peptide pattern in proteins to develop new antibiotics is a valid drug design strategy, but its relevance to explaining the physiology of extinct animals might require clarification. While the primary use of identifying peptide patterns within protein sequences for drug design is distinct from the challenges of explaining extinct animal physiology, the analysis of these patterns can still offer valuable, albeit limited, information about the physiological capabilities of extinct organisms. A deeper understanding of their biological context is necessary to fully appreciate the multifaceted roles the ancient molecules may have played in extinct species.^3,32,58,59^

### In Search of Neanderthal Cathelicidins

Neanderthals possessed cathelicidins, a family of antimicrobial peptides, similar to those found in humans. These peptides, like human cathelicidin LL-37, are part of the innate immune system and play a role in defending against infections.^60^ Cathelicidins are ancient and common participants of vertebrate innate immunity, identified in multitude of vertebrate species including all mammals.^61–63^ They are characterized by a conserved proregion and a highly variable antimicrobial peptide domain. Scientists are exploring the potential of Neanderthal cathelicidins as sources of novel antibiotics.^64^ They developed a machine learning model that could mine proteomic and genomic data from Neanderthals and Denisovans. The model finds sequences from archaic humans and predicts which ones would be good antibiotic candidates.^2,39,65^

### Paleoproteomic Methodologies of Molecular De-extinction

Paleoproteomics, the study of ancient proteins, provides a critical foundation for the molecular de-extinction, involving mining the proteomes of extinct organisms, thus providing a direct window into the molecular biology of extinct species and enabling the recovery, sequencing, and functional characterization of their proteins (Figure 3). This approach utilizes machine learning and other computational methods to identify and analyze these molecules, with the goal of discovering new drugs or therapies.^2,32^ Unlike ancient DNA, proteins are more chemically stable, surviving in specimens millions of years old. By combining paleoproteomic data with modern synthetic biology, researchers can reconstruct and test the functions of proteins lost to extinction.

**Figure 3:**
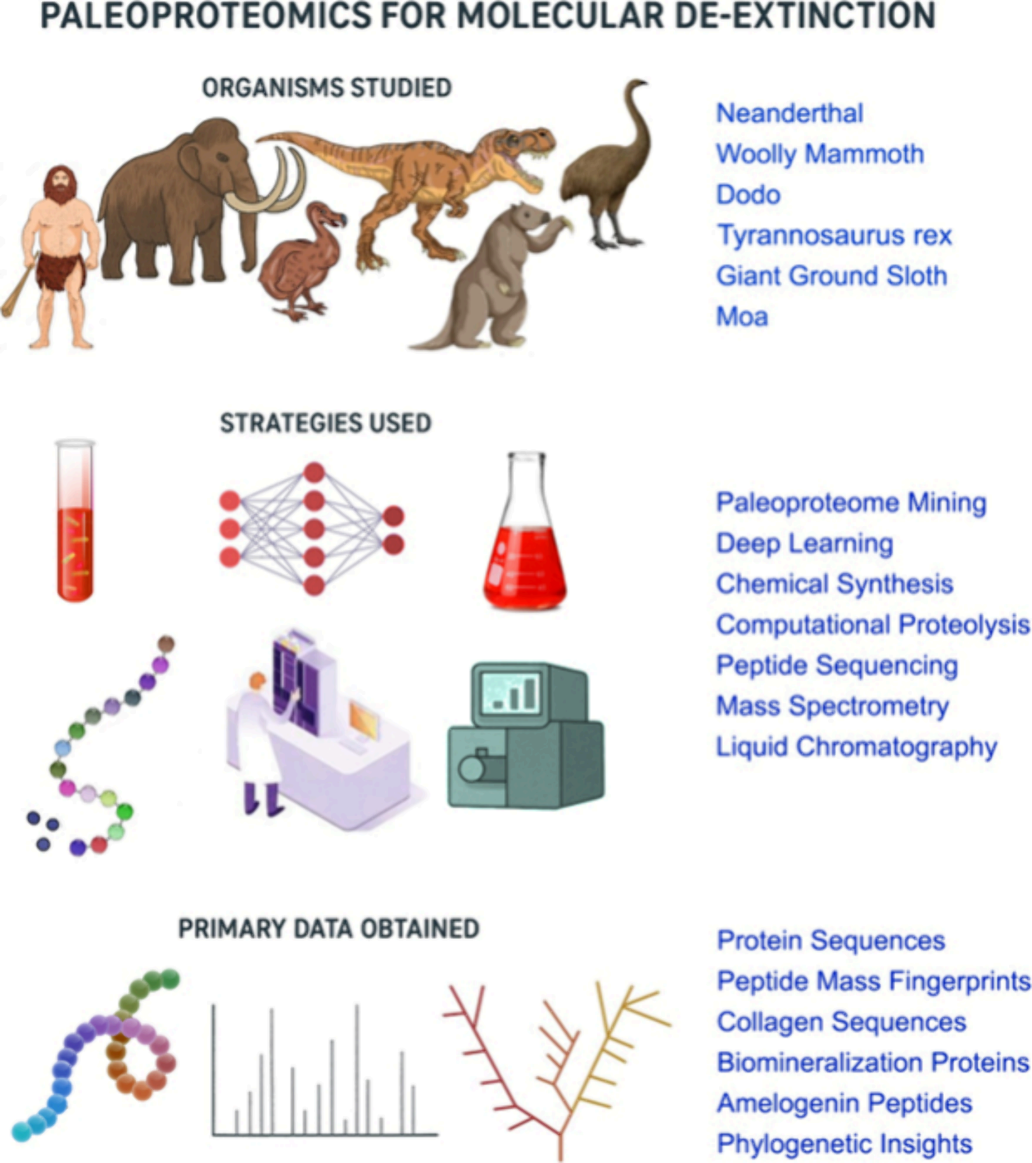
Scheme of the paleoproteomics approach in de-extinction, illustrating the organisms studied, the strategies, and the primary data obtained.

Molecular de-extinction via paleoproteomics involves the extraction, sequencing, computational reconstruction, and functional resurrection of proteins from extinct organisms. This methodology leverages advances in mass spectrometry, bioinformatics, and synthetic biology to recover and study ancient biomolecules.

The paleoproteomic pipeline for molecular de-extinction includes:

- ***Fossil selection and protein preservation***. Preservation depends on: taphonomic conditions (temperature, pH, mineralization); tissue type (bone, tooth enamel, eggshells are most stable); geological age (proteins degrade over time but can persist for >1 million years). Screening techniques: zooarcheology by mass spectrometry (ZooMS)—rapid collagen fingerprinting to identify species; immunoassays—antibody-based detection of surviving protein fragments.^66^
- ***Protein extraction and sequencing***. Extraction methods include: acid digestion (e.g., HCl) to solubilize collagen; guanidine-HCl or urea for noncollagenous proteins; nondestructive methods (e.g., EDTA demineralization) for rare specimens. Mass spectrometry (MS) analysis: liquid chromatography–tandem MS (LC-MS/MS)—identifies peptide sequences; high-resolution MS (Orbitrap, TOF)—enhances detection of degraded peptides; de novo sequencing—critical when reference genomes are unavailable.^67–69^

- ***Bioinformatic reconstruction***. Since ancient proteins are often fragmented, computational tools are used to align sequences to extant homologues (BLAST, HMMER); predict 3D structures (AlphaFold2, Rosetta, I-TASSER, and the recent addition of AlphaFold 3 representing a significant step change in the field of molecular biology and protein structure prediction); model functional dynamics (Molecular Dynamics simulations).^70–75^

- ***Synthetic biology resurrection***. Gene synthesis—the inferred protein sequence is codon-optimized and synthesized; heterologous expression—produced in *E. coli*, yeast, or mammalian cells; functional assays—test enzymatic activity, ligand binding, or structural properties.^76–78^

### Barriers to Clinical Translation and Potential Solutions

Antimicrobial peptides (AMPs), often derived from proteolytic cleavage, hold promise as novel therapeutics to combat antibiotic resistance. However, their clinical translation is hindered by instability (susceptibility to protease degradation), potential toxicity (e.g., cytotoxicity or hemolysis), delivery challenges (difficulty penetrating biological barriers such as biofilms), and poor bioavailability (limited solubility or rapid clearance). Certain chemical strategies and nanotechnological approaches have been developed to overcome these barriers to clinical translation, enhancing the pharmacokinetic and pharmacodynamic properties of AMPs.^2,32,37^

Chemical strategies such as peptidomimetics, cyclization, conjugation, and sequence optimization enhance AMP stability and reduce toxicity by modifying their structure. Nanotechnological approaches, including nanoparticle encapsulation, nanoemulsions, surface-modified carriers, and nanogels, improve delivery, bioavailability, and targeting.^79–81^ Combining these—e.g., peptidomimetics in nanoparticles—shows significant promise, with studies reporting substantial bacterial load reductions *in vivo*. Continued research into cost-effective synthesis, scalability, and long-term safety will be critical to realizing the clinical potential of AMPs.^82–86^

## 4 Paleogenomics

### Defensins Identified through Molecular De-extinction

Defensins are small, disulfide-rich cationic peptides that play a vital role in the defense mechanisms of living organisms, especially in host immunity. Eight extinct vertebrate genomes have been computationally mined searching for defensins and examining their evolution and structure.^37^ Six authentic β-defensins have been identified as a result, five of which are derived from two different extinct bird species and one from a mammalian species (Table 3).^3,37^ These organisms included an extinct moa species (*Anomalopteryx didiformis*) that inhabited New Zealand and the extinct Spix’s macaw (*Cyanopsitta spixii*), which was endemic to Brazil, as well as the black rhino (*Diceros bicornis minor*).^3,33,37,87^ Evolutionary and structural analyses of the β-defensins are performed to further characterize these molecules. This study identifies molecules from extinct organisms, opening new avenues for antibiotic discovery. Moreover, by examining structural information, including the secondary structure, cysteine motifs, disulfide bonds, tertiary structure similarities, and precursor gene sequence, a better understanding of their evolutionary relationships can be achieved.

**Table 3:** Representative Extinct β-Defensins Identified by Machine Learning but without Validating Their Activity Experimentally

Extinct species	β-defensin names	Peptide sequence
*Anomalopteryx didiformis*	*Ad*-AvBD5	TRQDCESRGGFCSRGSCPLGITRIGICSLQDFCCRRKMGE
	*Ad*-AvBD10	VSFADTEECRSQGNFCRPVSCPPVFSVSGSCYGGAMKCCKKEYGQ
*Cyanopsitta spixii*	*Cs*-AvBD1	NKAQCHREKGFCALLKCPFPYVISGRCTKFTFCCKKGA
	Cs-AvBD10	DPLFPDTTECKNQGNFCRAGTCPPTFAISGSCHGGLLRCCSKKISS
	*Cs*-AvBD9	PAYSQVDADTAACRQNRGSCSFVECSSPMVNIGTCRSGKLKCCKXYV
*Diceros bicornis minor*	*Db*-BD4	SSCHRNGGRCLLFVCFPGKTLIGNCGFPGSRCCR

Here, certain clarification of terms is needed. Molecular de-extinction is a scientific field that generally aims to resurrect molecules that are no longer encoded by living organisms to address current challenges, such as antibiotic resistance. It focuses on identifying, synthesizing, and understanding the biological functions of these ancient molecules by exploring evolutionary history recorded in molecules like nucleic acids and proteins.^31^ The difference between molecular de-extinction and molecular de-encryption, within the context of the described work, lies in the source of the molecules: **Molecular de-encryption** (or encrypted peptide prospection) involves discovering fragments (peptides) with antimicrobial properties within existing protein sequences that are cleaved from larger proteins. These peptides are “encrypted” within the protein and released through proteolytic cleavage. While the protein itself might be considered “extinct” if the organism is extinct, the focus is on these encrypted peptide fragments. **Molecular de-extinction**, in a more canonical sense, refers to the resurrection of molecules that are completely extinct, meaning they are no longer encoded by any living organism. The work by Ferreira et al.^37^ searching for defensins in extinct organisms represents this more canonical example because they aimed to identify naturally occurring defense molecules (defensins) derived from extinct organisms, thereby illustrating the resurrection of molecules that no longer exist in extant life. Their work demonstrates that molecular de-extinction can be used to find and study complete, naturally occurring defense molecules, representing a more canonical example of this approach.

### Paleomycin, the Ancestor of Modern-Day Glycopeptide Antibiotics

To shed light on the characteristics of ancestral glycopeptide antibiotics (GPA)—such as vancomycin, ristomycin and teicoplanin—and inform the development of future drugs, researchers combined bioinformatics with genetic and biochemical techniques to trace their evolutionary roots, even bringing to life the hypothesized precursor antibiotic, “paleomycin”.^38,88,89^ They expected that paleomycin would be a complex molecule, resembling the modern GPA teicoplanin. First, the nonribosomal peptide synthetase assembly line of paleomycin was predicted, and a guide tree based on biosynthetic gene clusters was constructed. Subsequently, by employing synthetic biology techniques, the predicted peptide was reconstructed and its antibiotic activity was validated.

During their evolutionary journey, major genetic shifts, including the merging, removal, or relocation of genes within their ancestral biosynthetic gene clusters, ultimately led to the creation of vancomycin-like GPAs, which are seen as simpler in structure. The study demonstrated how combining computational techniques with synthetic biology methods can reveal the temporal evolution of antibiotics and enable the resurrection of ancestral molecules. The insights gained into nature’s optimization tactics for modern GPA evolution can inform future efforts to engineer this crucial class of antibiotics.

### Paleogenomic Methodologies of Molecular De-extinction

The idea of de-extinction has transitioned from science fiction to a tangible scientific pursuit, thanks largely to advances in paleogenomics—the study of ancient DNA (aDNA).^42–44^ While whole-organism de-extinction (e.g., cloning a mammoth) remains controversial and technically challenging, molecular de-extinction offers a more feasible alternative: the resurrection of specific genes, proteins, or metabolic pathways from extinct species. Molecular de-extinction of metabolic pathways in this context refers to the scientific concept of reintroducing or recreating the molecular components and processes that constituted metabolic pathways in extinct organisms. Essentially, it is about bringing back the “recipes” for how extinct life forms transformed matter and energy. In simpler terms: researchers are using ancient DNA to find the “ingredients” and “instructions” for important biological processes that are no longer present in living organisms. This approach has already yielded functional insights into evolutionary biology, such as the cold-adaptation mechanisms of Pleistocene megafauna; the neurogenetic differences between modern humans and Neanderthals; the immune system evolution of extinct pathogens (Figure 4). Here we explore the paleogenomic pipeline for aDNA recovery and analysis as well as the computational and synthetic biology methods for gene resurrection.

**Figure 4:**
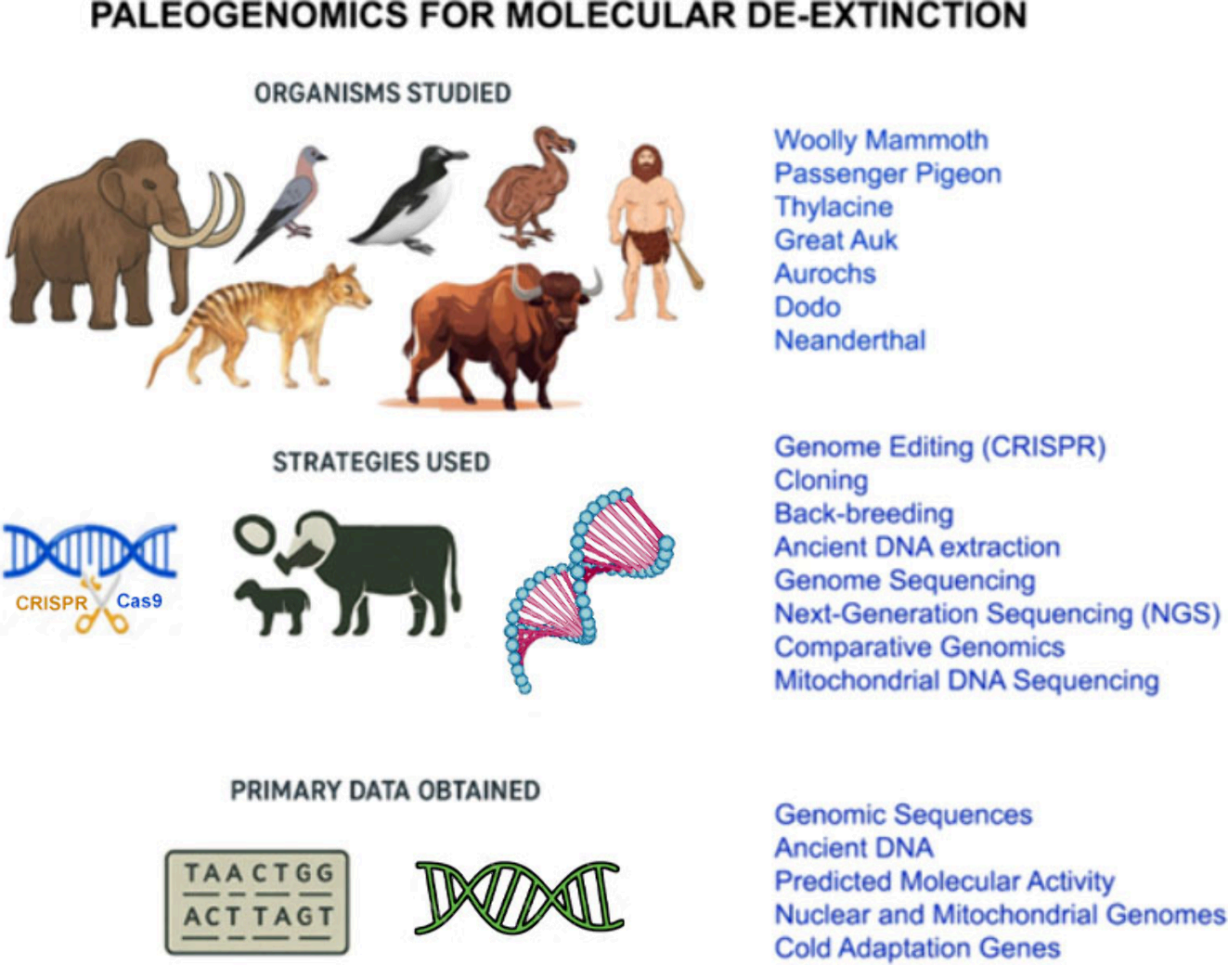
Scheme of the paleogenomics approach in de-extinction, illustrating the organisms studied, the strategies, and the primary data obtained.

#### Ancient DNA Extraction and Sequencing

Paleogenomic approach to molecular de-extinction aims to revive extinct species by reconstructing their genomes and introducing them into closely related living organisms. The first and most crucial step in this process is obtaining high-quality ancient DNA from preserved biological material, including sample collection, DNA isolation, next-generation sequencing (NGS), and computational genome assembly. Unlike modern DNA, aDNA is highly degraded, chemically modified, and often contaminated with microbial and environmental DNA. Advances in DNA extraction, sequencing technologies, and bioinformatics have made it possible to recover and analyze aDNA, paving the way for de-extinction efforts such as the woolly mammoth and thylacine projects.^90–92^

Ancient DNA can be extracted from a variety of sources, including: (i) Permafrost-preserved specimens (e.g., woolly mammoths, Pleistocene horses); (ii) Museum specimens (e.g., skins, bones, feathers of passenger pigeons, dodos); (iii) Subfossils (e.g., cave bear bones, moa eggshells); (iv) Dried remains (e.g., mummified tissues, herbarium samples). Factors affecting DNA preservation include: temperature—cold, dry environments (permafrost, caves) best preserve DNA; pH—neutral or slightly alkaline conditions reduce DNA degradation; microbial activity—high microbial presence accelerates decay; time—older samples (>1 million years) are often too degraded for recovery.^93–95^

## 5 Synthetic Biology in De-extinction

### Genome Editing

While ancient DNA (aDNA) extraction and sequencing provide the blueprint, genome editing and synthetic biology are the tools that bring these blueprints to life.^96^ Unlike cloning, which requires intact nuclei, genome editing allows scientists to modify the DNA of living species to resemble their extinct relatives. This approach has been central to projects targeting the woolly mammoth, passenger pigeon, and thylacine.^97–99^

Therefore, the resurrection of extinct species through molecular de-extinction relies heavily on genome editing and synthetic biology. These technologies enable precise modifications to the genomes of closely related living species, introducing extinct traits to recreate functional proxies of lost organisms. Key methodologies include CRISPR-Cas9 gene editing, synthetic DNA reconstruction, stem cell engineering, and interspecies genome hybridization. Technical barriers include: (i) incomplete genomes—gaps in ancient DNA sequences require computational prediction; (ii) gene regulation—epigenetic and noncoding DNA elements are poorly preserved; (iii) off-target effects—unintended mutations can disrupt development.^1,97,100^

### CRISPR-Cas9 and Precision Gene Editing

The CRISPR-Cas9 system uses a guide RNA to direct Cas9 nuclease to specific DNA sequences, enabling targeted cuts and modifications. It has been applied in woolly mammoth de-extinction: Asian elephant genomes are edited to include mammoth genes for cold adaptation (e.g., hemoglobin, fat storage, and hair growth). Another exemplary application is in the Passenger Pigeon de-extinction: band-tailed pigeon genomes are modified to restore extinct traits like flocking behavior and coloration. Advantages of the method are its high precision, relatively low cost, and scalability.^101–103^

### Base and Prime Editing

Base editing converts one DNA base pair to another without double-strand breaks (e.g., C → T or A → G transitions). It is useful for correcting point mutations in extinct genomes. Prime Editing allows for small insertions, deletions, and all possible base changes with minimal off-target effects.^104–106^

### Multiplex Genome Engineering

In simultaneous edits, multiple genes can be edited in a single step, crucial for complex traits such as mammoth cold adaptation. Challenges of the method are its off-target effects and unintended disruptions to gene regulation.^107,108^

### Synthetic DNA Reconstruction

De novo gene synthesis: when ancient DNA is too degraded, synthetic biology can reconstruct genes based on computational predictions. The method has been applied in the thylacine genes—synthetic versions of thylacine immune and skeletal genes are inserted into fat-tailed dunnart cells. It has been applied also in the Woolly Mammoth Traits—Artificial versions of mammoth-specific alleles are synthesized and tested in elephant cells.^76,77,109^

### Artificial Chromosome Engineering

Whole-genome synthesis: Large DNA fragments or entire chromosomes can be synthesized and inserted into host cells. The challenge of the method includes Ensuring proper chromosomal integration and gene expression.^110,111^

### Surrogate Species and Embryo Development

While genome editing provides the genetic blueprint for de-extinction, successful species revival ultimately depends on producing viable offspring. Most de-extinction projects require surrogate hosts from closely related extant species to carry edited embryos to term. This stage presents numerous challenges including reproductive compatibility, embryo-maternal interactions, and developmental synchronization between species. Recent advances in assisted reproductive technologies (ART) and stem cell engineering are helping overcome these barriers, bringing de-extinction closer to reality.^112,113^

Thus, the final critical stage of molecular de-extinction involves developing edited embryos through surrogate species. This process presents unique biological and technical challenges requiring careful selection of host species, advanced reproductive technologies, and innovative solutions for gestation. It involves methodologies for surrogate selection, embryo engineering, interspecies pregnancy management, and alternative development systems.

### Cellular Reprogramming and Cloning: Stem Cell Engineering and Interspecies Chimera-Induced Pluripotent Stem Cells (iPSCs)

Somatic cells from living relatives (e.g., Asian elephants) are reprogrammed and converted to iPSCs, which can then be edited. The advantage of this approach is that it enables unlimited cell proliferation and differentiation into various tissue types.

### Interspecies Blastocyst Complementation

Edited stem cells are injected into early embryos of a surrogate species, creating chimeric organisms. Example: Mammoth-like elephant stem cells can be introduced into Asian elephant embryos.

### *Ex Utero* Development and Artificial Wombs

Some species (e.g., thylacines) lack close living relatives for natural gestation. Artificial womb technology is being developed for the ectogenesis of edited embryos.

### Challenges and Ethical Considerations

Reconstructing complete genomes from degraded DNA is difficult, and hybrids may not fully replicate extinct species. Unintended genetic consequences could also arise from the editing of complex genomes. Ecological risks should be considered—reintroducing species could disrupt modern ecosystems, introducing competition or disease. Synthetic biology must account for how resurrected species interact with current environments. Ethical dilemmas exist as well—critics argue that de-extinction diverts resources from conserving living species. Questions also arise about the welfare of hybrid organisms and whether they belong in today’s world.

In general, de-extinction raises several ethical considerations, including animal welfare, environmental impact, and potential unintended consequences. While arguments in favor of de-extinction such as restoring ecosystems and fostering conservation efforts exist, others express concerns about the welfare of resurrected animals as well as the possibility of disrupting existing ecosystems. Indeed, there are worries about the potential suffering of resurrected animals, as they may not be well-suited to their reintroduced environment. De-extinction could disrupt existing ecosystems, cause competition with native species, and lead to unforeseen ecological consequences. The unknown immunogenicity of ancient molecules is also a concern. Some argue that de-extinction is a form of overstepping the boundaries of human interference in natural processes. So, the ethical considerations surrounding de-extinction are complex and multifaceted, and they will require careful consideration and ongoing dialogue as the technology continues to develop.^25,114,115^

## 6 Medical Impact Prospects, Challenges, and Future Directions

### Medical Impact Prospects

De-extinction has traditionally been associated with restoring extinct species, such as the woolly mammoth or passenger pigeon. However, a more immediate and impactful application lies in molecular deextinction—the resurrection of extinct genes, proteins, and biochemical pathways for medical use.

Ancient organisms evolved under different environmental pressures, leading to unique molecular adaptations that could address modern medical challenges. By leveraging advances in ancient DNA sequencing, computational biology, and protein engineering, scientists can reconstruct and test these molecules for their therapeutic potential.

Antimicrobial resistance (AMR) is a global health crisis, with traditional antibiotics becoming increasingly ineffective. One of the most promising applications of molecular de-extinction is the discovery of novel antimicrobial agents.^2,3,18,36^ Ancient organisms produced antimicrobial peptides that may offer novel mechanisms of action against resistant pathogens, suggesting that prehistoric molecules may bypass modern resistance mechanisms and offer new therapeutic avenues. Noteworthy, antibiotic resistance is not solely a result of modern antibiotic overuse. Bacteria have been producing and competing with antibiotic-like substances since ancient times. Resistance mechanisms, like those found in ancient cave bacteria, likely evolved as a way to survive in their environment long before humans started using antibiotics clinically.^116,117^

Vaccine development using ancient pathogens: resurrected viral epitopes—extinct viruses (e.g., 1918 influenza) can be studied to predict future pandemic strains and develop broad-spectrum vaccines; paleovirology insights—ancient viral sequences in genomes can reveal conserved targets for vaccine design.^118,119^

Anticancer and antiaging properties: elephant TP53 genes: extinct proboscideans had multiple copies of tumor suppressor genes (e.g., TP53), which could be adapted for cancer therapy; longevity genes from extinct species—some extinct species exhibited extreme longevity; their genetic adaptations could inform antiaging research.^120–122^

Some extinct species had highly effective immune responses. For example, ancient retroviral defenses—endogenous retroviruses (ERVs) in extinct mammals may hold clues for blocking modern retroviral infections like HIV. This suggests potential applications in vaccine adjuvants (enhancing immune responses); possible use in autoimmune disease therapy (due to evolutionary differences in immune regulation).^123–125^

### Challenges and Limitations

Molecular deextinction—the process of reconstructing and reintroducing extinct genes, proteins, or metabolic pathways—holds immense promise for medicine, biotechnology, and evolutionary biology. However, significant scientific, technical, ethical, and ecological challenges must be addressed before this technology can be widely applied.

- DNA degradation and incomplete genomic data, including: (i) fragmentation—ancient DNA (aDNA) is often damaged, making full gene reconstruction difficult; (ii) contamination—microbial and environmental DNA can obscure target sequences; (iii) epigenetic loss—methylation and other regulatory elements are rarely preserved, affecting gene expression. Potential solutions include advanced computational modeling, including techniques such as machine learning (ML) and large language models (LLMs), and comparative genomics (using closely related extant species as templates). Noteworthy, training models and algorithms are going to be crucial, and this may also lead to bias due to the use of modern DNA for training. This bias can affect downstream analyses and potentially lead to some truly ancient genetic information being lost or misinterpreted, as it might not be accurately represented in the modern-based reference.^126,127^

- Functional uncertainty of resurrected molecules, including: (i) protein folding errors—resurrected proteins may misfold due to differences in cellular environments; (ii) post-translational modifications—ancient proteins might require extinct enzymes for proper function; (iii) toxicity or immunogenicity—modern organisms may reject or react adversely to ancient biomolecules.
- Delivery and integration into modern systems issues include: (i) gene silencing—host organisms may suppress foreign genes; (ii) off-target effects—CRISPR edits could disrupt essential genes; (iii) horizontal gene transfer risks—engineered genes could spread uncontrollably in ecosystems.
- Unknown immunogenicity: Could ancient peptides trigger adverse immune responses?
- Ecological impact: Releasing revived microbes risks unintended consequences.
- Bioethics: Should extinct organisms’ biomolecules be commercialized?

Economic and logistical barriers include: (i) high costs–older DNA extraction, synthesis, and testing require substantial funding; (ii) regulatory hurdles–longer approval processes for medical or environmental applications; and public skepticism–the fear of “playing God” may limit funding and acceptance. Potential solutions may include public engagement to demystify science as well as government and private-sector partnerships. It is worth reminding that The European Court of Justice (ECJ) ruled in 2018 that organisms developed through new mutagenesis techniques, including gene editing methods like CRISPR, are considered genetically modified organisms (GMOs), falling under the same strict GMO regulations, including required environmental and food/feed risk assessments.^128,129^ The idea of using gene editing to release ancient organisms faces a greater uphill battle due to the ethical and environmental concerns already present in the gene editing debate.

## 7 Conclusion and Future Directions

Molecular de-extinction is advancing rapidly due to breakthroughs in DNA sequencing, gene editing, and computational biology. While initial efforts have focused on reconstructing individual genes (e.g., antimicrobial peptides from extinct species), future research will likely expand into broader applications, including precision medicine (personalized therapies based on ancient genetic adaptations), synthetic biology (designing hybrid organisms with extinct traits), and biotechnology (using ancient enzymes for industrial processes).

Key future directions may include:

- AI and machine learning in ancient protein reconstruction: predictive modeling—AI can simulate how extinct proteins fold and function, bypassing the need for complete DNA sequences; deep learning for gene synthesis—neural networks can predict missing fragments in degraded ancient DNA, improving reconstruction accuracy; automated drug discovery—AI can screen thousands of resurrected molecules for potential therapeutic effects.
- CRISPR and gene drives for functional resurrection: precision editing—CRISPR-Cas9 and base editing can “humanize” ancient genes for safe medical use; gene drives for disease resistance—extinct immune genes could be reintroduced into modern species (e.g., malaria-resistant mosquitoes); xenotransplantation—resurrected genes from extinct animals (e.g., mammoth hemoglobin) could improve organ preservation.
- Synthetic biology and hybrid organisms: chimeric cell lines—combining extinct genes with modern cells to study disease resistance (e.g., Neanderthal immune genes in human cell cultures); engineered microbiomes—using ancient gut bacteria to treat modern metabolic disorders; synthetic organelles—resurrecting extinct cellular machinery for biofuel production or waste degradation.
- Ecological and environmental applications: de-extinct enzymes for bioremediation—ancient microbes could break down modern pollutants (e.g., plastic-degrading enzymes from extinct bacteria); climate adaptation genes—resurrecting cold-resistant genes from Pleistocene megafauna to help crops withstand climate change.
- Ethical and regulatory evolution: global biosecurity policies—preventing misuse of de-extinction technology (e.g., weaponized pathogens); indigenous rights and genetic sovereignty—ensuring communities retain control over ancestral genetic material; patent law and ownership—establishing frameworks for commercializing extinct genes without monopolization.

Molecular de-extinction, the science of resurrecting extinct genes and proteins, represents a paradigm shift in antibiotic discovery, offering a unique reservoir of unexploited antimicrobial potential. While challenges remain in scaling and regulation, early successes demonstrate that Earth’s lost biodiversity may hold the key to solving the antimicrobial resistance crisis. Strategic integration of paleogenomics, AI, and synthetic biology could soon make “paleoantibiotics” a frontline defense against superbugs.

## Supplementary Material



## Notes

Figure S1. Documents related to key concepts associated with the molecular de-extinction, as found in the CAS Content Collection (PDF)
