# Supplementary material for: Molecular Paleontology Meets Drug Discovery: The Case for De-extinct Antimicrobials
Source: ACS Omega. 2025 Sep 10;10(37):42175–87. doi: 10.1021/acsomega.5c05530 (PMC12461407; doi:10.1021/acsomega.5c05530)
Supplement: Supplementary file 1 [file ao5c05530_si_001.pdf]

Supporting Information

Molecular Paleontology Meets Drug Discovery: The Case for De-Extinct Antimicrobials

Rumiana Tenchov, Qiongqiong Angela Zhou*

CAS, a division of the American Chemical Society, Columbus OH 43210, USA

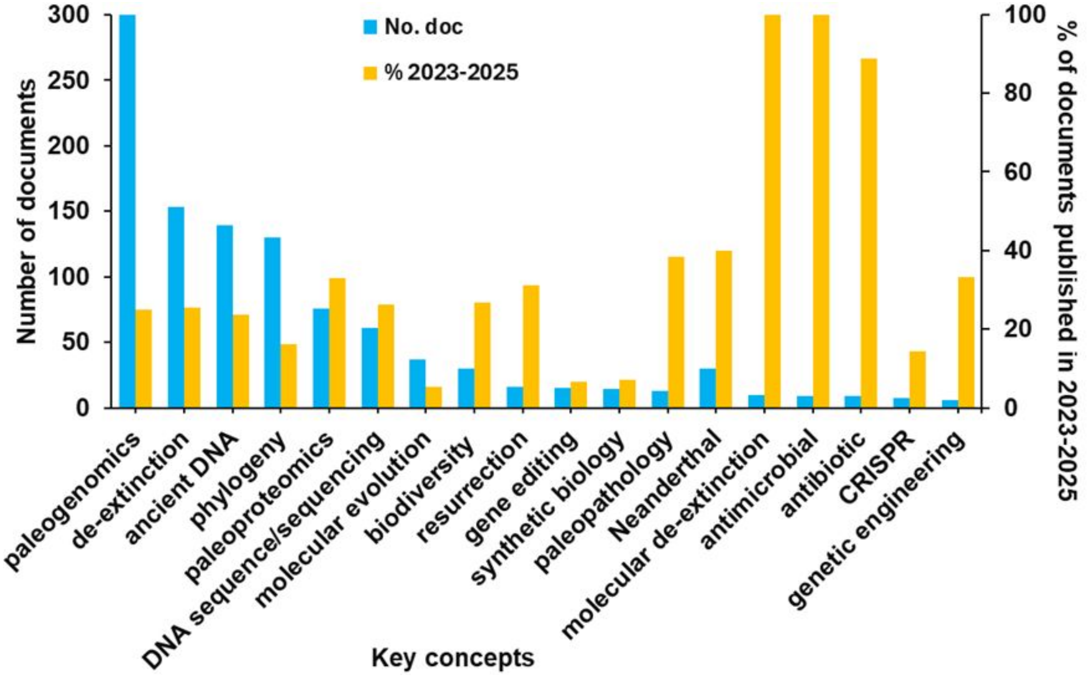

Figure S1. Documents related to key concepts related to the molecular de-extinction, as found in the CAS Content Collection: Overall number of documents (blue bars, left Y-axis; Percentage of documents published in 2023-2025 (yellow bars, right Y-axis; data for 2025 only through March).
